# Meeting Report: Development of Environmental Health Indicators in Brazil and Other Countries in the Americas

**DOI:** 10.1289/ehp.8486

**Published:** 2005-11-16

**Authors:** Fernando F. Carneiro, Mara Lúcia C. Oliveira, Guilherme F. Netto, Luis A.C. Galvão, Jacira A. Cancio, Estela M. Bonini, Carlos F. Corvalan, the Participants in the International Symposium on the Development of Indicators for Environmental Health Integrated Management

**Affiliations:** 1 General Coordination for Environmental Health Surveillance in Health Surveillance, Secretariat/Ministry of Health of Brazil, Brasília, Brazil; 2 Pan-American Health Organization, Office in Brazil, Brasília, Brazil; 3 Pan-American Health Organization, Environmental Health and Sustainable Development, Washington, DC, USA; 4 Catholic University, Brasília, Brazil; 5 World Health Organization, Geneva, Switzerland

**Keywords:** environmental health indicators, environmental health surveillance, integrated management

## Abstract

This report summarizes the Brazilian experience on the design and implementation of environmental health, with contributions from Argentina, Canada, and Cuba, presented at the International Symposium on the Development of Indicators for Environmental Health Integrated Management, held in Recife, Pernambuco, Brazil, on 17–18 June 2004. The methodology for the development of environmental health indicators has been used as a reference in the implementation of environmental health surveillance in Brazil. This methodology has provided tools and processes to facilitate the understanding and to measure the determinants of risks to environmental health, to help decision makers control those risks.

From 1998 to the present, the Ministry of Health of Brazil has implemented environmental health surveillance (EHS) within the National Public Health System. The methodology for the development of environmental health indicators became one of the key issues for the implementation of EHS. The driving force, pressure, state/situation, exposure, effects, action (DPSEEA) model approach was applied to develop appropriate indicators (Corvalan et al. 1999). This matrix model takes into consideration the relationship cycle process among economic and social dynamics, environmental response, and human health. Thus, the driving forces consist of the economic and social processes, resulting in pressures such as the intensive use of particular natural resources. Both driving forces and pressures contribute to the situation/state scenarios where the environment is often contaminated or deteriorated, facilitating human exposure to environmental risk factors that might produce effects on health. For each of these categories, indicators and action proposals are developed to gain a more complete understanding of the problem as well as to visualize measures to be taken in each level of complexity of the cycle (Figure 1).

## The Regional Experience

An international symposium on the development of indicators for integrated environmental health management, as a prior activity to the VI Brazilian Congress of Epidemiology, took place in Recife, Brazil, on 17–18 June 2004. The symposium involved presentation and discussions on the Brazilian experience with the development of environmental health indicators and exchange of experiences with similar initiatives in Canada, Cuba, and Argentina. The symposium was organized by the Ministry of Health of Brazil under the auspices of the Pan American Health Organization/World Health Organization and the Associação Brasileira de Pós-Graduação em Saúde Coletiva’s Health and Environment Working Group.

The main objectives of the symposium were to assess the use of the indicators for the environmental health integrated management in the countries of the Americas, to present the Brazilian experience in the development of indicators regarding the integrated management of environmental health, and to contribute, through the development of environmental health indicators, to the strengthening of the following initiatives: *a*) the Health and Environment Ministers of the Americas meeting, *b*) the Pan American Health Organization Environmental Health Collaborating Centers Network, and *c*) methodologies for the integrated assessment of environmental health in the Americas.

The experiences in developing indicators in some countries in the American continents were evaluated, with particular attention to their usefulness in the decision-making processes. A summary of the indicators developed and evaluated are shown in Table 1, and national and international examples of the application of indicators are described in Table 2.

## Conclusions and Recommendations

The symposium, in addition to presenting the experiences in Brazil and other countries, assessed ways of adopting the proposed methodology for the development of environmental health indicators and their use in the integrated management of actions on related health and environment issues, with the following conclusions:

- The development of the environmental health indicators should be interdisciplinary and participatory, focused on the economic, social, and environmental contexts that represent risk situations to human health.
- The indicators should be built based on priorities, considering what society considers to be a problem, and should lead to health protection and the promotion of changes, as well as to facilitate decision making, considering health as a social value.
- The development of environmental health indicators should also respect and incorporate community-based knowledge, the interdisciplinary and intersectorial approach toward integrated management.
- The participation of government decision makers in the identification and development of indicators is highly valuable because it results in an improved future management of the problems identified.

The environmental health indicators matrix (DPSEEA) also proved useful for the analysis of complex problems and for the analysis of emerging issues, such as the products of nanotechnology, genetic manipulation, and nonionizing radiation among others.

DPSEEA, which is a framework that organizes information from economic and social dynamics, environmental response, and human health, addresses all the complex levels of a given problem, and it is more efficient in the analysis of such problems compared with other cause-and-effect models. It promotes a holistic view of the issues and is therefore effective in the identification of relevant indicators. It is also a valuable tool for learning, training, and deeper discussions on complex exposure–effect process relationships in environmental health.

Workshop participants commented that DPSEEA would have some limitations if understood only in a linear and vertical form without considering the complex socioenvironmental contexts and their relation with underlying health problems, environment, and quality of life.

## Figures and Tables

**Figure 1:**
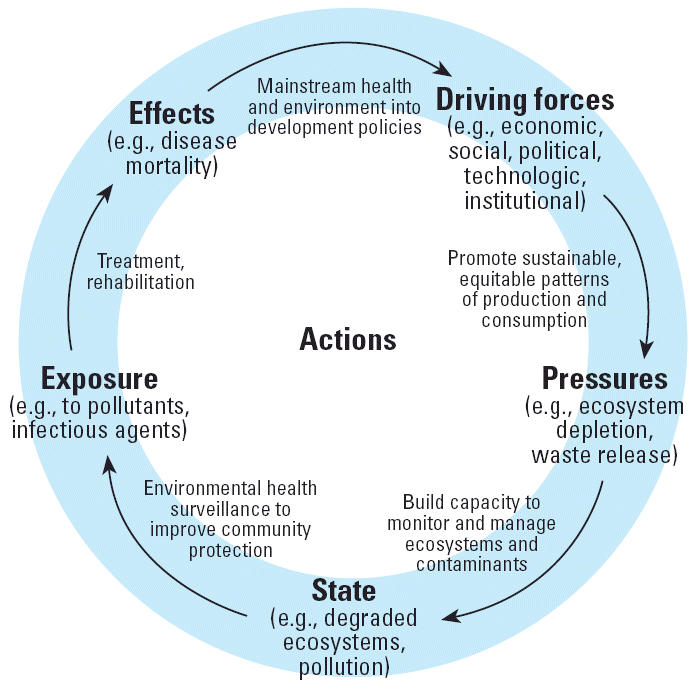
The DPSEEA model (adapted from Corvalan et al. 2000).

**Table 1:** Some examples of environmental health indicators in use, selected from applying the DPSEEA matrix to different situations.

Country/topics	Indicators developed	Matrix categories
Brazil	Per capita consumption	Pressure
Water (Maciel Filho et al. 1999)	Water bacteriologic quality	Exposure
	Residual chlorine	Exposure
	Water supply services coverage	Situation
	Water supply regularity	Situation
Cuba	Economic policy of healthy residences development	Driving force
Healthy residences (Placeres MR et al., unpublished data)	Percent of resources for the construction of healthy residences	Pressure
	Percent of households with appropriate ventilation	Situation
	Percent of households with appropriate lighting	Situation
	Percent of households with appropriate kitchen installations	Situation
Canada	Release of substances that damage the ozone layer	Pressure
Ultraviolet radiation (Bartlett S, unpublished data)	Ozone layer reduction rate	Exposure
	Cumulative radiation doses	Exposure
	Ultraviolet radiation indicators	Exposure
	Incidence of melanoma	Effects
	Eye damage	Effects

**Table 2:** National and international experiences in the use of indicators.

Country	Source	Program	Description
Brazil	Guimarães MJB, Melo NGDO, Lima AAF, Camarão F, Fillho JAN, Lyra TM, unpublished data	EHS drinking water program	Good example of the use of environmental health indicators for decision making: Ministry of Health developed database allowing local-level data entry accessible on line at municipal, state, and federal levels
			Database can potentially be analyzed together with mortality and morbidity data: Successes include drinking water risk maps generated by the Municipality of Recife that served as a base to restructure and reinforce health surveillance actions to priority areas
			Recognized as a useful application and given an award by the Health Surveillance National Secretariat
Cuba	Placeres MR, Rojas MC, Diaz VIP, Toste MA, Melián CMG, unpublished data	DPSEEA	Has been used in the family health program, orienting doctors and nurses to consider social, political, economic, and geographic factors in identifying environmental problems that affect people’s health
			Has oriented the establishment of the environmental primary health care strategy by choosing environmental health local indicators to achieve an integration of local health development
Canada	Bartlett S, unpublished data	Environment Canada	Has been reporting on environmental indicators since 1990 in its National Environmental Indicator Series and State of the Environment reports
			In the Environment and Sustainable Development Indicators initiative’s final report, the National Round Table on the Environment and the Economy (NRTEE) proposed six new indicators, developed in collaboration with Environment Canada and Statistics Canada (released May 2003): forest cover, freshwater quality, air quality, greenhouse gas emissions, extent of wetlands, and educational attainment
		Health Canada	Uses the DPSEEA framework to organize the environmental health indicators
			Currently collaborating with Environment Canada on the development of an air–health indicator (AHI)
			The potential AHI builds on ongoing work on improved air quality index to develop a multipollutant trend indicator that directly links potential health impacts to exposure to pollution, as recommended by NRTEE
			Such an indicator has also helped to assess the effectiveness of pollution mitigation strategies and public health interventions
